# Case of Protracted Labor

**Published:** 1871-08

**Authors:** L. A. Harcourt

**Affiliations:** Chicago


ART. II.—*Case of Protracted Labor.* By L. A. HARCOURT, M. D., Chicago.

---

At 11 o'clock on the night of Nov. 30th, 1870, I was summoned to attend Mrs. King, Elston Road, Chicago, aged 27 years, in labor with her first child. At 2 p. m. the day before, while engaged in rinsing clothes, she was suddenly seized with pain in her right side, which compelled her to go to bed. Since that time she had had pains, recurring at irregular intervals of 10, 20 or 30 minutes, sometimes ceasing altogether for an hour. She had not kept her bed all the time; but had slept none since the pains came on. She did not expect to be confined before the 15th of December.

An examination per vaginum showed that labor had begun, but made little progress. The os was thick, rigid and undilated, far back towards the sacrum, and so high up that it could barely be reached by the index finger. The vertex presented, over which the anterior portion of the inferior segment of the uterus was drawn like a night-cap. Through this segment the sagittal suture could be distinctly felt, occupying the left oblique diameter of the pelvis, the posterior fontanelle at or near the left acetabular region. The pelvis was below the normal size, without deformity of contour, and as the woman herself was small, the probabilities were that the size of the fœtal head would be commensurate with that of the pelvis, hence I did not anticipate trouble on that score. The pulse was normal, respiration easy, bowels and kidneys in good condition, yet the woman looked a little anxious. Told her her labor would be tedious, but nevertheless safe, to borrow no trouble and all would be well. I then went home, leaving two or three Dover's powders for her to take with a view of procuring sleep.

At 7 a. m. Dec. 1st, called again. The patient had taken only one powder, and had slept none. The pains had continued all night to very little purpose. The os was slightly dilated, but still unyielding; patient fatigued and worn from want of rest. Prescribed the chloral hydrate in ten grain doses, to be repeated every two hours until rest would be obtained. At 1 o'clock p. m. the patient had slept comfortably for two hours, also between the pains. The os was still rigid, dilatation very slight. Prescribed tart. Ant. et Potass in minute doses, combined with morphia, to be repeated every hour; the chloral to be given as required.

At 7 p. m. called again, and found the patient comfortable. She had slept all the afternoon except during the pains. Her bowels had moved spontaneously, and the bladder had been freely evacuated. The rigidity of the os had very much disappeared, the dilatation being about the size of a dollar. The pains were more efficient, and the prospect seemed fair that the labor would terminate that night. In fact there was no apparent reason why it should not be completed by midnight. Remained all night, and had an excellent opportunity of observing the effects of the chloral hydrate, which I administered from time to time. The patient would wake up during a pain, and the instant it was over, fall into a natural and refreshing sleep. Thus it continued all night, and though the labor progressed slowly, I had the satisfaction of knowing that my patient had gained fresh strength and vigor to renew the conflict on the coming day. Just here, it may not be out of place to say that in cases of this kind, I have found the hydrate of chloral a most admirable remedy. Have used it where opium would not be tolerated, and in every instance with the happiest results.

At 7 a. m. Dec. 2d, went home to breakfast. Called three or four times in the course of the day to note the progress of the case. It was very tedious, but as the membranes were still intact, I concluded to exercise patience, and not interfere unless the safety of mother or child demanded intervention.

At 8 p. m. the dilatation being almost complete, and the os no longer rigid, I ruptured the membranes, hoping that the uterus would contract more firmly, and thus expedite the delivery. The pains did increase, still the progress was very slow. By ten o'clock rotation had taken place, and the occiput began to engage in the arch of the pubes. The uterine efforts seeming ineffectual, I gave half a teaspoonful of the fluid extract of ergot. In fifteen minutes gave a teaspoonful, and in half an hour more gave two teaspoonfuls, and so on until the patient had taken in all an ounce, without producing the desired result, viz., vigorous and expulsive pains.

At half-past twelve the head ceased to advance. It was not actually locked, but filled the pelvic cavity like a wedge, and I became convinced that nature would not accomplish the delivery in time to save the child, if, indeed, it succeeded at all. The woman, too, could not continue the struggle much longer without seriously compromising her own safety. Not having my forceps with me, and living a long way from the place, I sent for Dr. R. H. Harcourt, who lived nearer. He applied the instruments, and in a few minutes delivered the woman of a healthy, living child. Her labor had continued almost uninterrupted for eighty-four hours, yet nothing untoward supervened. Her recovery was speedy and complete. I attributed her comparative exemption from exhaustion, the usual result of protracted labor, to the rest obtained from the judicious use of the chloral hydrate.

